# Supplementary material for: Disentangling metabolic functions of bacteria in the honey bee gut
Source: PLoS Biol. 2017 Dec 12;15(12):e2003467. doi: 10.1371/journal.pbio.2003467 (PMC5726620; doi:10.1371/journal.pbio.2003467)
Supplement: S8 Data — (ZIP) [file pbio.2003467.s008.zip › Z-score_plots/585 microbial substrate 4-(beta-D-Glucosyloxy)benzoate.pdf]

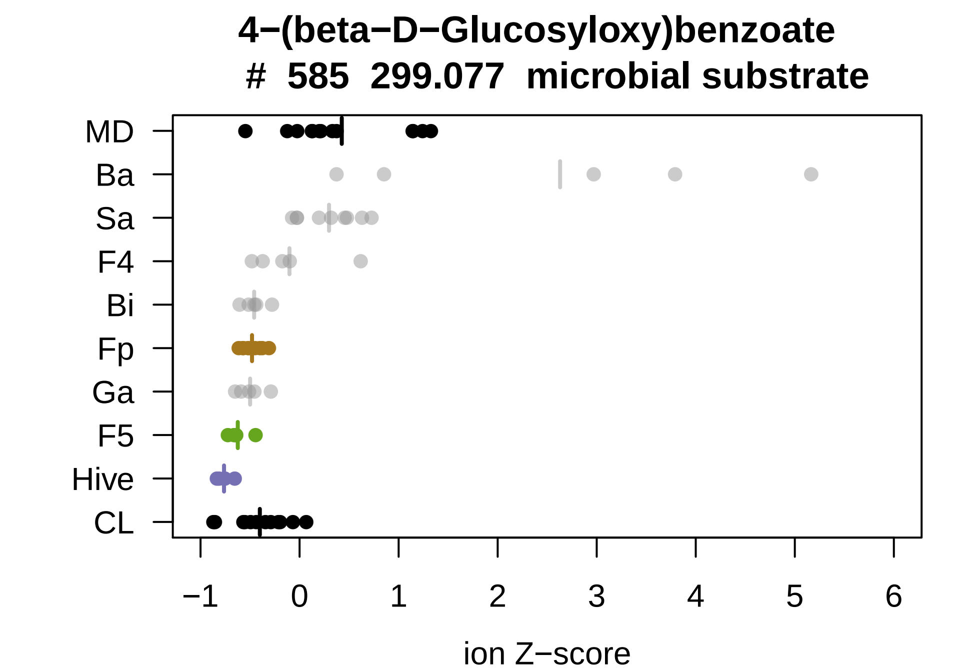
4−(beta−D−Glucosyloxy)benzoate
585 299.077 microbial substrate
MD
Ba
Sa
F4
Bi
Fp
Ga
F5
Hive
CL
−1
0
1
2
3
4
5
6
ion Z−score
